# Azo dying of α‐keratin material improves microbial keratinase screening and standardization

**DOI:** 10.1111/1751-7915.13541

**Published:** 2020-02-28

**Authors:** Milena Gonzalo, Roall Espersen, Waleed A. Al‐Soud, Francesco Cristiano Falco, Per Hägglund, Søren J. Sørensen, Birte Svensson, Samuel Jacquiod

**Affiliations:** ^1^ Section of Microbiology University of Copenhagen DK‐2100 Copenhagen Denmark; ^2^ Department of Biotechnology and Biomedicine Technical University of Denmark DK‐2800 Lyngby Denmark; ^3^ Department of Chemical and Biochemical Engineering Technical University of Denmark DK‐2800 Lyngby Denmark; ^4^ Present address: Interactions Arbres/Micro‐organismes INRA/Univ. de Lorraine Champenoux France; ^5^ Present address: Department of Biomedical Sciences Panum Institute 12.6 University of Copenhagen Copenhagen Denmark; ^6^ Present address: Agroécologie AgroSup Dijon INRAE Univ. Bourgogne Univ. Bourgogne Franche‐Comté F‐21000 Dijon France

## Abstract

Microbial conversion through enzymatic reactions has received a lot of attention as a cost‐effective and environmentally friendly way to recover amino acids and short peptides from keratin materials. However, accurate assessment of microbial keratinase activity is not straightforward, and current available methods lack sensitivity and standardization. Here, we suggest an optimized Azokeratin assay, with substrate generated directly from azo‐dyed raw keratin material. We introduced supernatant filtration in the protocol for optimal stopping of keratinase reactions instead of the widely used trichloroacetic acid (TCA), as it generated biases and impacted the sensitivity. We furthermore suggest a method for standardization of keratinase activity signals using proteinase K, a well‐known keratinase, as a reference enabling reproducibility between studies. Lastly, we evaluated our developed method with several bacterial isolates through benchmarking against a commercial assay (Keratin Azure). Under different setups, the Azokeratin method was more sensitive than commonly used Keratin Azure‐based assays (3‐fold). We argue that this method could be applied with any type of keratin substrate, enabling more robust and sensitive results which can be used for further comparison with other studies, thus representing an important progress within the field of microbial keratin degradation.

## Graphical abstract

We introduce ‘Azokeratin’, an optimized protocol significantly increasing sensitivity for screening of microbial keratinases.

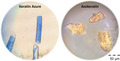

## Introduction

Livestock industries produce vast amounts of keratin‐based waste (e.g. feathers, bristles, wool, hooves and horns). For instance, worldwide poultry slaughterhouses process around 50 billion chickens per year, and 5%–7% of the total weight consists of feathers (Kozák, 2011). In the case of pork meat production, a crucial economic sector in Denmark, 22 million pigs are slaughtered each year on average (Danish Crown Group, 2016), resulting in accumulation of substantial amounts of low‐value by‐products requiring proper disposal. Indeed, as each slaughtered pig generates around one kilogram of bristles and hooves, ~22 000 tons of keratin‐based by‐products are handled yearly in Denmark. Being classified as category‐3 waste by European agencies, cautious handling of keratin‐based pig by‐products is required, as they may pose environmental and sanitary risks (Korniłłowicz‐Kowalska and Bohacz, 2011; Verma *et al.*, 2017).

Keratin materials are generally insoluble, recalcitrant and highly heterogeneous with significant variation in terms of protein conformation (e.g. α‐helix and β‐sheets), supramolecular 3D assembly (e.g. layer and filament structures) and biochemical composition (amino acid content and disulphide bond arrangement; Bragulla and Homberger, 2009). Keratin fibers are tightly packed and cross‐linked through disulphide bridges, conferring high mechanical stability and resistance to well‐known proteases such as papain, trypsin and pepsin (Gupta *et al.*, 2013). There are, however, several mechanisms to degrade keratinous materials, and controlled microbiological‐mediated processes are an alternative for valorization of keratin by‐products by conversion into amino acids and short peptides (Korniłłowicz‐Kowalska and Bohacz, 2011; Gupta *et al.*, 2013; Kang *et al.*, 2018).

Indeed, keratin biodegradation may be achieved *via* microbial enzyme cocktails (Lange *et al.*, 2016) containing both proteolytic and disulphide cleavage capabilities. The proteolytic action is performed by specific proteases, termed keratinases (Lin *et al.*, 1992; Onifade *et al.*, 1998; Rozs *et al.*, 2001; Brouta *et al.*, 2002), while the disulphide opening can occur by reduction or sulfitolysis (Onifade *et al.*, 1998). In this field of research, accurate assaying of the keratinolytic potential is crucial to understand the degradation process, and detect new microorganisms/enzymes of interest for downstream applications.

Indeed, innovative screening methods and activity assays are always needed to improve detection sensitivity and discovery of novel enzymes for many applications (Jacquiod *et al.*, 2014; Ufarté *et al.*, 2016). Nevertheless, the field of keratin degradation still lacks effective detection procedures, as assays must be adequately adapted to the targeted keratin substrate and relevant keratinase families involved. This is mainly due to the fact that keratin proteins exist as either α‐keratins (α‐helix, wool, bristles, hairs and hooves) or β‐keratins (β‐sheet, feathers, beaks and bird claws; Gopinath *et al.*, 2015). Although the use of azo dying has been reported for assaying β‐keratin degradation (Jeevana Lakshmi *et al.*, 2013; Pereira *et al.*, 2014), numerous publications still rely on Keratin Azure, a blue‐dyed sheep wool made of α‐keratin, to monitor β‐keratin degradation (e.g. feathers, Gupta and Ramnani, 2006; Ningthoujam *et al.*, 2016). Considering the popularity of Keratin Azure usage in the literature, we question its relevance as a ‘broad screening method’ since it may be suboptimal in some cases.

Additionally, assay standardization is still lacking in order to obtain reproducible keratin‐degradation measurements. Most published data involve absorbance recordings as a proxy for keratinase activity, yet, these values cannot be readily translated between studies (Gupta and Ramnani, 2006). This significantly hampers the field by limiting comparability and reproducibility of results generated under different setups. External calibration using varying amounts of a known enzyme with chromophore‐labelled authentic substrates is a well‐known method for assaying degradation of recalcitrant biopolymers (Jacquiod *et al.*, 2013). However, to the best of our knowledge, this procedure was never applied to keratin degradation.

We argue that, due to heterogeneity of existing keratin substrates, a relevant approach consists of using directly the investigated material itself as substrate for enzymatic assays, making it ideal for accuracy and reproducibility reasons. Here we introduce a comparison between two methods for screening of α‐keratin degradation, namely the only available and widely used Keratin Azure commercial assay, and our present strategy using Azokeratin prepared directly with the targeted substrate, in this case α‐keratin from pig slaughterhouse by‐products (e.g. bristles and hooves). The underlying goal is to define better strategies to handle this potentially important bioresources, notably through microbial bioconversion (Kang *et al.*, 2018). Furthermore, going beyond mere measuring of chromophore absorbance values, the present study represents the first attempt to systematically standardize keratinolytic activity using external calibration with purified proteinase K (a well‐known, accessible and widely used keratinase). We optimized our assay from the initial established procedure (Lin *et al.*, 1992) by removing the use of trichloroacetic acid (TCA) as a reaction‐stopping reagent, replacing it by a vacuum‐manifold filtration system. For testing, we used three bacterial strains known to possess different keratinolytic profiles: (i) *Bacillus licheniformis*, one of the most efficient industrially applied bacteria in feather degradation (Gupta *et al.*, 2013) that has a serine protease‐type keratinase (Lin *et al.*, 1992), (ii) *Bacillus subtilis* (Stefanic and Mandic‐Mulec, 2009) which is a soil strain containing a metallo‐keratinase (Tork *et al.*, 2013) and finally, (iii) an unknown Gammaproteobacterium isolate related to *Stenotrophomonas *spp. obtained from a mesophilic keratin enrichment culture that had high proteinase and keratinase activities (Fig. S1).

## Results and discussion

### Relationship between pH and absorbance spectra of chromogenic keratin products

**Figure 1:**
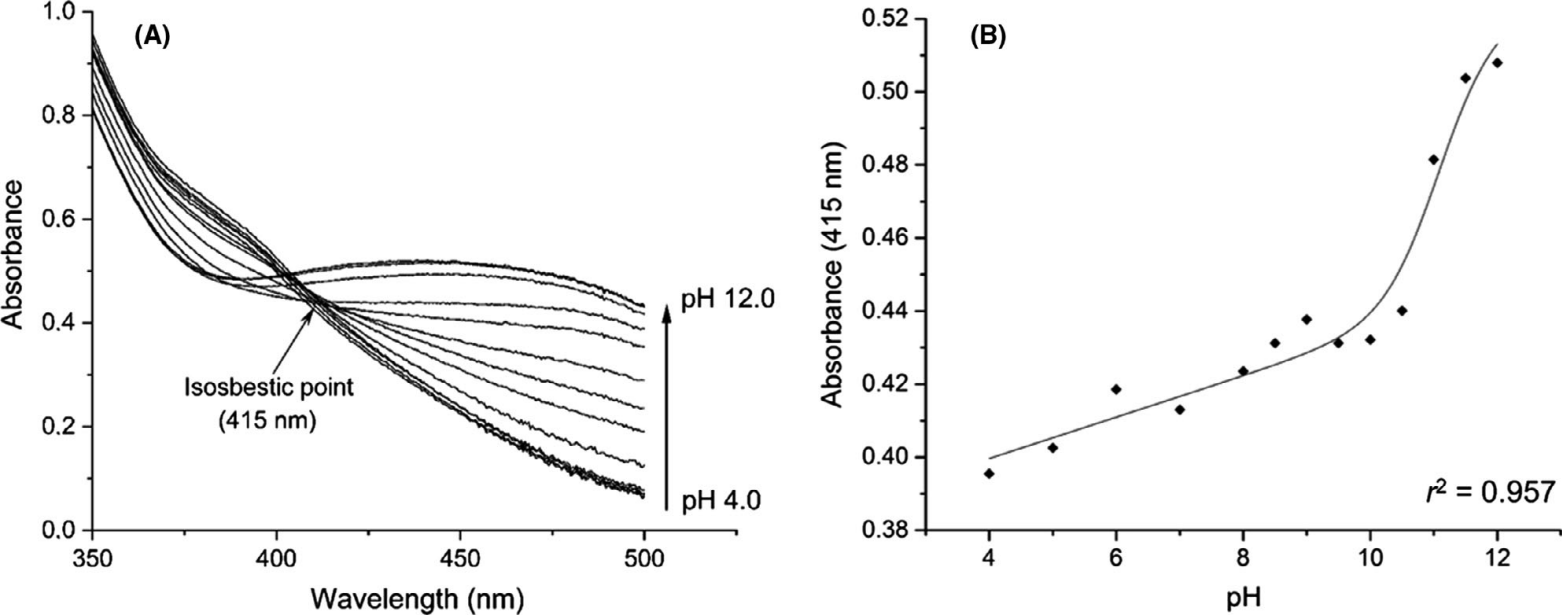
Effect of pH on spectral properties of products released from Azokeratin. A. The spectra (350–500 nm) of Azokeratin products recorded in the pH range 4–12. The thick arrow indicates the spectra with increasing pH. It is seen that the signal at 450 nm, the conventional wavelength for product quantification, is very dependent on pH. An isosbestic point is observed around 415 nm (thin arrow). B. Absorbance at 415 nm is relatively stable at pH 4–10 pH. Above pH 10 the azo‐chromophore is deprotonated giving rise to an increase in signal.

First, the link between pH and absorbance spectra of pig keratin‐degradation products was investigated to decide whether using NaOH for signal stabilization could be avoided. It was observed that spectra of the dyed keratin‐degradation products, obtained with proteinase K, are pH‐dependent, indicating differential protonation levels (Fig. 1A). At the commonly used wavelength (450 nm), acidification lowered the signal, leading to a colour shift from orange/red to paler yellow, thus demonstrating a strong correlation with pH. This change, however, can be overcome by adding NaOH after removal of undigested substrate to regain signal at higher pH, revealing titration of the azo‐chromophore. Spectral scans indicated an isosbestic point around 415 nm at pH 4–10 (Fig. 1A); hence, measurements at this wavelength are much less sensitive to pH fluctuations compared with 450 nm, eliminating the need for NaOH addition if the reaction is stopped without TCA (Fig. 1B). Thus, 415 nm was found to be the most suitable wavelength to measure soluble products in the Azokeratin assay.

Notably, products from Keratin Azure and Azokeratin behaved differently at lowered supernatant pH. Absorbance signal decreased sharply at low pH (< 4), accompanied by appearance of a blue precipitate corresponding to Keratin Azure degradation products (Fig. S2). The signal intensity was not recovered by NaOH addition, as products are removed from solution. Thus, the signal‐loss stems from precipitation of chromophore‐labelled products, not from a change in spectral properties by titration. This permanent intensity loss makes the Keratin Azure assay impractical when pH is ≤ 3.

### Comparison between filtration and TCA addition for ending the degradation process

**Figure 2:**
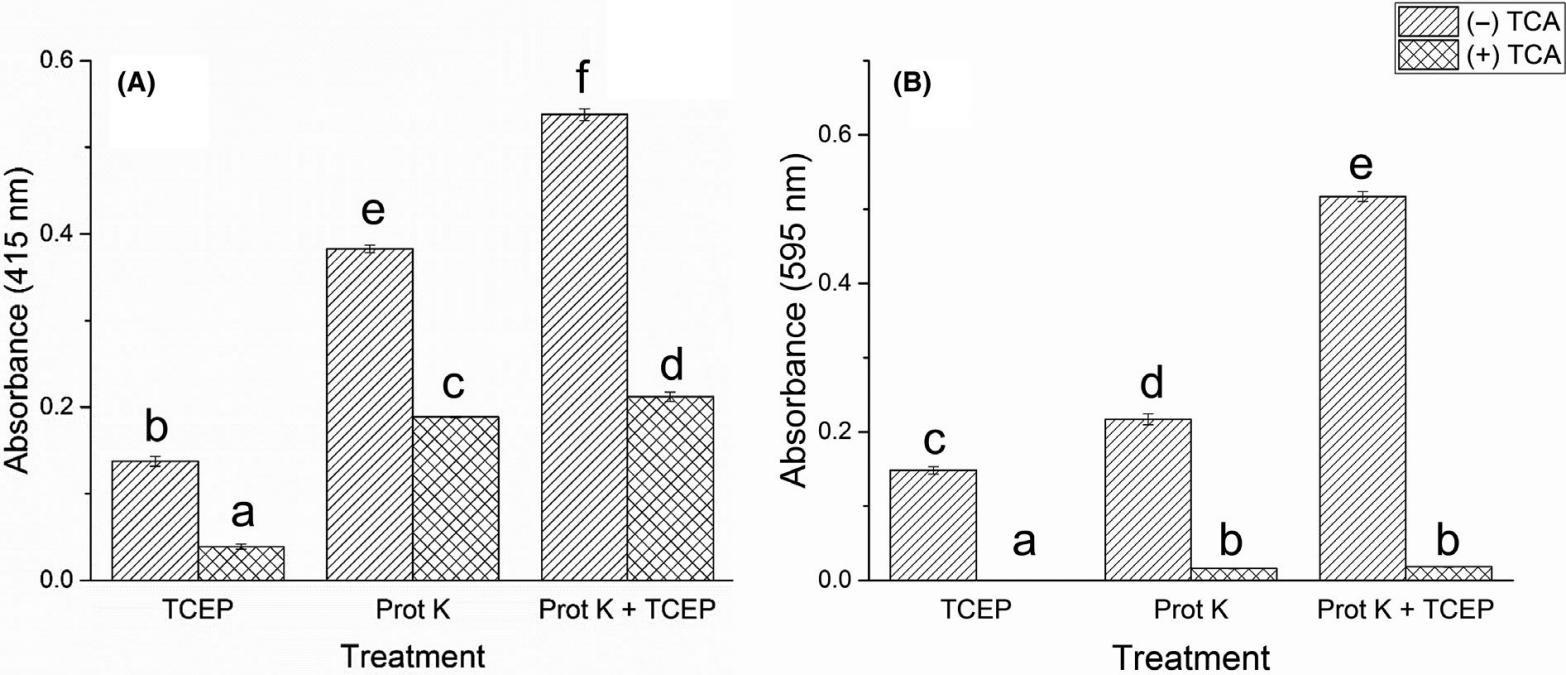
Comparison of signals from two different methods to stopping the degradation reaction for Azokeratin (A) and Keratin Azure (B). The two different substrates were treated using three different conditions, TCEP, Proteinase K (Prot K) and both proteinase K and TCEP (Prot K + TCEP) (A) Signal loss with Azokeratin as substrate is significant and at least above 50% for all three treatments. (B) The Keratin Azure is much more affected by TCA, and the signal loss is critically high. Statistical analysis was inferred using ANOVA implemented with post hoc Tukey test (*P* < 0.05).

Efficient on‐demand stopping of enzymatic reactions is crucial for getting accurate and reproducible results. Here, the use of TCA addition is compared with supernatant filtration for ending reactions. The absorbance was measured on (i) filtered supernatants and (ii) filtered and then TCA‐treated supernatants. First, the use of TCA resulted in significantly lowered signals with both substrates (*P* = 1.37E−3, Fig. 2), but the Keratin Azure products were by far the most affected (Fig. 2B). As shown above, Keratin Azure products were influenced by low pH that seemed reinforced by TCA, probably because peptides and proteins are precipitated in addition to sample acidification. These combined adverse effects caused drastic and almost complete loss of signal for the Keratin Azure assay. The precipitation renders the Keratin Azure assay about 20‐fold less sensitive when TCA is used to stop the reaction. The reason behind the distinct behaviour of the two types of dyed products at low pH, especially in the presence of TCA, is not known.

Meanwhile, the signal loss for Azokeratin was not as important (Fig. 2). We, however, investigated whether some of the products from filtered samples were precipitated by TCA, or if the signal drop was only due to pH change. Product precipitation would mean that signal intensity cannot be fully rescued after TCA addition by subsequent NaOH introduction. When comparing supernatants before/after TCA precipitation, the supernatant treated with TCA prior to addition of NaOH failed to recover signal intensity comparable to samples where NaOH was added before TCA (Fig. S3A). Equivalent signal is observed for a sample with NaOH added before TCA compared with one added NaOH and buffer instead of TCA (Fig. S3B).

Another striking observation was that the relative signal loss differed between degradation treatments, as it was much higher for samples treated with the disulphide reducing agent Tris(2‐carboxyethyl)phosphine (TCEP; Fig. S3A). TCEP is hypothesized to release products of larger molecular weight in the supernatant as no breakage of peptide bonds are involved as opposed to protease degradation. Precipitation of degradation products thus seems to depend on their nature, reflecting that TCA might precipitate polypeptides presumably released by TCEP with more efficiency. Indeed it has been reported that peptides of different sizes and physicochemical properties are precipitated by TCA to different extent (Yvon *et al.*, 1989). To further investigate this effect on the products, two *Amycolatopsis keratinophila* proteases purified from a culture supernatant (Espersen *et al.*, 2020) were analysed with the Azokeratin assay (Fig. S3A). TCA addition caused higher signal loss with both these proteases compared with proteinase K, while the *Amycolatopsis keratinophila* culture supernatant showed intermediate behaviour to proteinase K and the two proteases. Thus, TCA precipitated degradation products differently amongst the tested proteases as opposed to the filtration method. Additionally, very similar signal losses resulted from 2% and 10% TCA with the different proteases, indicating limited effect of TCA concentration (Fig. S4).

Considering the fundamental difference between assaying degradation of non‐soluble and soluble substrates, we argue that enhancing the signal from the soluble products obtained from insoluble keratin substrates leads to superior sensitivity with the filtering method rather than TCA acidification. This is in part due to the probable bias that TCA has towards precipitating larger peptides. Thus, a protease releasing larger peptides will present a disadvantage when utilizing TCA together with the Azokeratin assay compared with another releasing smaller peptides. Indeed, the TCA method was clearly sensitive to the kind of protease considered. Based on these results, we recommend using filtration instead of TCA to stop keratinolytic reactions, especially for the well‐known Keratin Azure assay, where it resulted in a gained ≈ 20‐fold increase in sensitivity. TCA is still the most utilized method for stopping enzymatic degradation of keratin (Zhang *et al.*, 2016; Su *et al.*, 2017) and was also considered for the development of a standard keratin substrate (Jin *et al.*, 2017). In rare occasions, heating (Wu *et al.*, 2017) and/or centrifugation (Habbeche *et al.*, 2014) may be used as reaction‐stopping alternatives. The use of filtration circumvents some of the short comings of TCA, like the bias for/against certain protease types, limiting the screening scope for discovering novel enzymes. It should be mentioned that the drawbacks observed using TCA, together with the substrate used in this study, will also apply to other keratin materials and detection approaches (e.g. ninhydrin assay and Bradford assay). Thus, our filtration‐based methodology should have broad appeal within the field of keratinolytic degradation.

### Proteinase K calibration of Azokeratin signal

**Figure 3:**
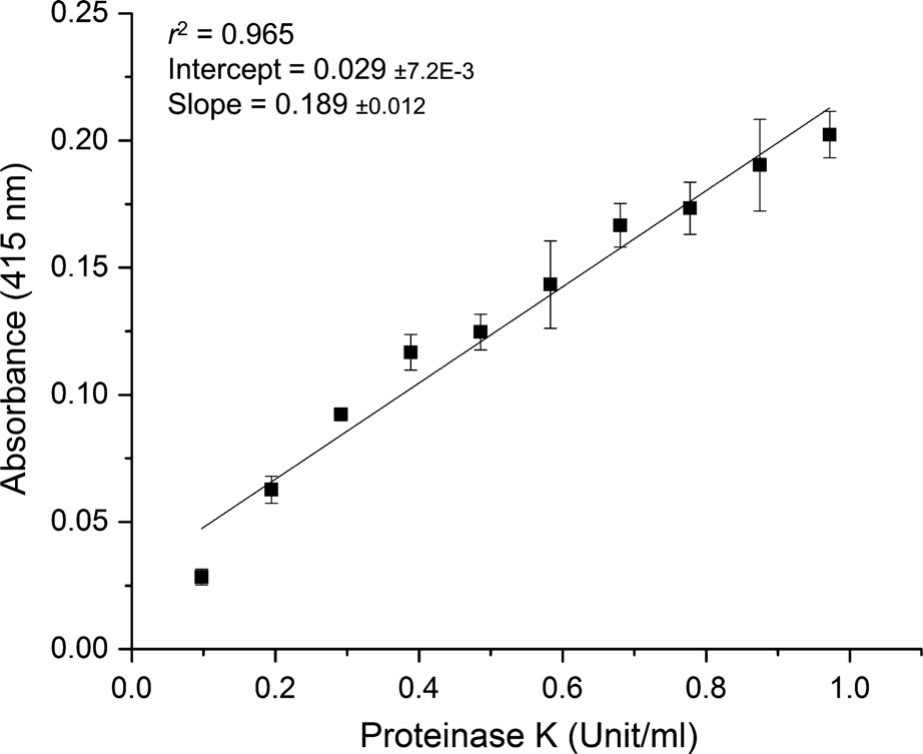
Proteinase K degradation of Azokeratin. The figure shows amount of released Azokeratin products with increasing proteinase K activity. Data points are fitted to a linear curve fit, which can be used to estimate the activity of an unknown sample relative to proteinase K.

To standardize the Azokeratin substrate, we utilized the activity of the well‐known proteinase K. The activity of proteinase K itself was first standardized with the synthetic substrate SPAN (*N*‐succinyl‐L‐phenylalanine p‐nitroanilide) (Fig. S5A). SPAN was selected as a well‐defined substrate that proteinase K is able to use, and for its homogeneity compared to keratin substrates, making it suitable for estimating the amount of active proteinase K in a stock solution. The trend of Azokeratin degradation (5 mg ml^−1^) with proteinase K (0.972 U) was linear as monitored by soluble Azokeratin products at 415 nm, indicative of a steady state reaction (Fig. S5B). It was estimated that either increasing the Azokeratin or decreasing the proteinase K concentration can still result in a linear release using 60 min incubation. Keeping the amount of active proteinase K constant (0.972 U) and varying the Azokeratin concentration gave a Michaelis–Menten‐like curve (*K*_M_ = 15 mg ml^−1^, Fig. S5C) and it was decided to use 25 mg ml^−1^ Azokeratin for calibration with proteinase K, as this would give both (i) a higher signal and (ii) a convenient Azokeratin concentration for practical handling reasons. The apparent *K*_M_ towards the keratin substrate reflects how much substrate protein is available in the keratin batch. Variability between keratin batch (e.g. origin and level of milling) can influence the amount of substrate available for degradation. A possible further level of standardization could be to always use the amount of keratin substrate equal to the *K*_M_ value or if possible, at much higher concentrations so that all assayed proteases are working at maximum capacity. The proteinase K activity varied between 0.0972‒0.972 U and data were fitted to a linear regression (*r* ^2^ = 0.9692; Fig. 3). This standard curve can be used to describe our Azokeratin batch with regard to both product availability and labelling efficiency.

Keratin by‐products are highly heterogeneous and azo‐labelling may not be equally efficient from batch to batch due to microscopic shape and size differences (Fig. S6). However, calibration with proteinase K provides standardization and enables data comparison from various substrate batches and between different studies. Currently, the common way for keratinase activity characterization and standardization is to divide signal intensity obtained from a chromophore‐labelled keratinous substrate (e.g. Keratin Azure or Azokeratin) with the signal achieved using a broad‐specificity protease substrate, for example Azocasein (K:C). This signal ratio is proposed to be > 0.5 for a keratinase (Evans *et al.*, 2000; Gupta *et al.*, 2013) although it depends heavily on substrate labelling and availability. Thus, if a given keratin substrate is poorly labelled or with low availability, the ratio will be lowered, resulting in high uncertainty and apparent lack of specificity towards keratin. Gupta *et al. *(2013) previously discussed the comparability problem by highlighting the large variation in K:C ratios reported for keratinases, and additionally, the variation in substrates used for its calculation (Gupta *et al.*, 2013). The presently proposed Azokeratin assay provides consistency, reproducibility and enables comparison between different studies. It is based on the keratinase‐like proteinase K being assayed accurately using a synthetic substrate, which in turn is used to standardize a batch of keratin substrate with regard to the amount of signal generated. This method would thus facilitate comparison between different studies, even if different substrate targets are used, as the targeted keratinase activity will be related to that of proteinase K as a way of standardization.

### Azokeratin assay testing on bacterial strains

**Figure 4:**
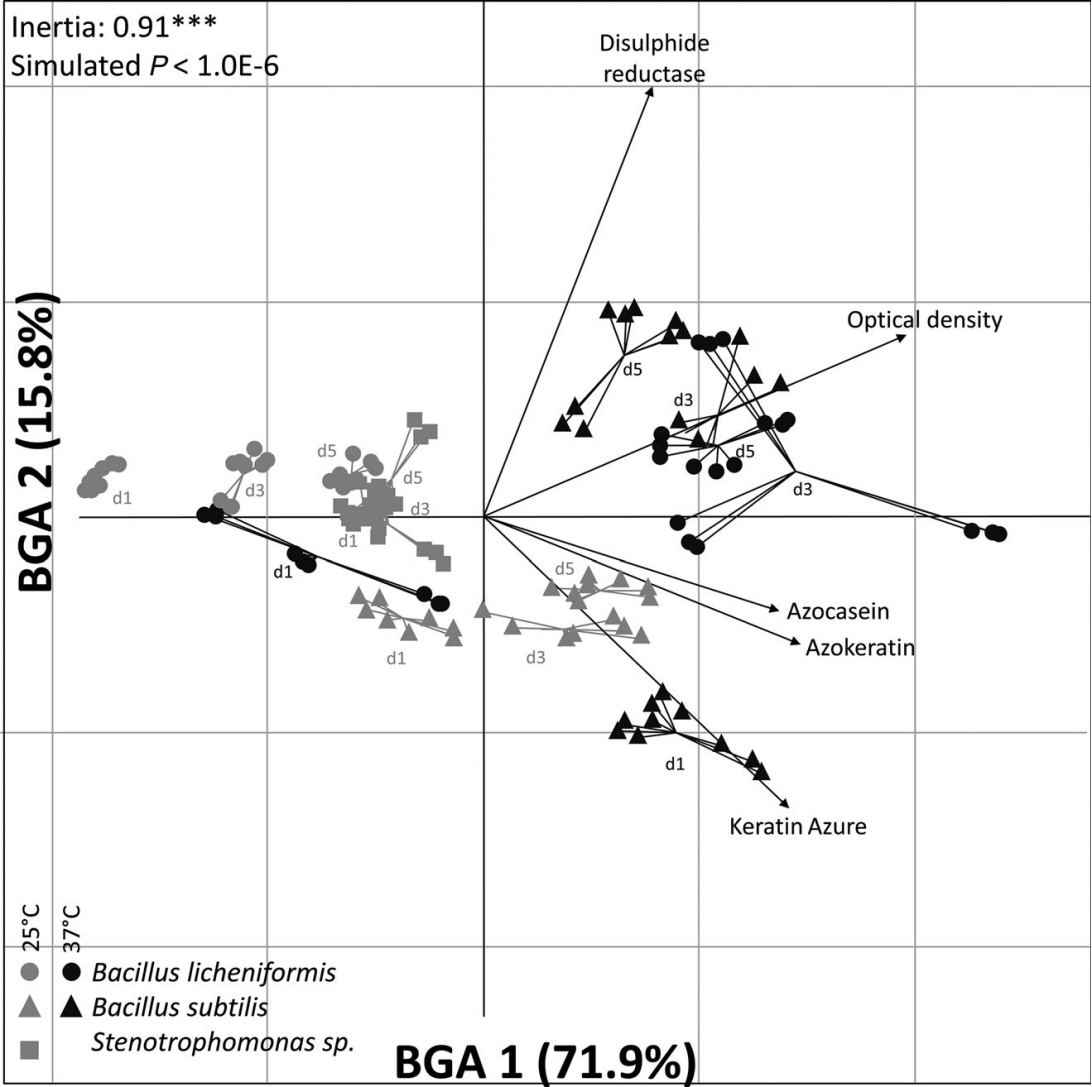
Between‐group analysis (BGA) applied on a principal component analysis (PCA) of the strain proteolytic profiles. This analysis includes Keratin Azure, Azokeratin, Azocasein, growth and disulphide reductase for *Bacillus licheniformis*, *Bacillus subtilis* and *Stenotrophomonas* spp. at 25°C and 37°C over time. (D stands for day; d1: day 1, d3: day 3, d5: day 5). Significance of replicate grouping was tested with a Monte Carlo simulation (*P* < 1.0E−6, ***)

To disentangle independent and combined factorial effects on the proteolytic profiles, a principal component analysis was performed, followed by a constrained analysis using a Monte Carlo simulation to test the significance of replicate grouping (Fig. 4). First, time and then temperature effects were clearly responsible for structuring the profiles, with segregation operating on both first (71.9%) and second components (15.8%). This separation was correlated with growth, keratinolytic and reductase activities. This is particularly true for both *Bacillus* strains and clearly indicates that all strains tested are using keratin as the sole nutrient source to support growth, but with very distinct patterns.

At 25 °C both *Bacillus* strains are showing gradual transitions according to sampling days, correlating with growth (Fig. 4). *Bacillus subtilis* (*Bs*) is clearly growing better than *Bacillus licheniformis* (*Bl*) which was correlating with keratinolytic activities. These differences persisted at 37°C, as *Bl* lags behind in terms of growth while *Bs* shows very strong correlation with keratinolytic activities after one day. Conversely, at 37°C, the interaction with sampling days was stronger, as a marked increase in activity occurred for both *Bacillus* strains between days one and three/five. Indeed, both strains shifted in a similar manner towards a combination of high keratinolytic and disulphide reductase activity alongside with increasing growth. Profiles at days three and five are very close, indicating that activity probably reached its highest level in terms of enzyme production and efficiency. These results are coherent with previous assessment of *in vitro* keratin degradation (Lange *et al.*, 2016), occurring after three/five days using chicken feathers meal with purified keratinase (Sanghvi *et al.*, 2016), pig bristles and hooves (Kang *et al.*, 2018; Falco *et al.*, 2019). This is also in accordance with degradation of other recalcitrant biopolymers (e.g. chitin), where an activity peak was observed after three/five days in pure culture (Kim and Ji, 2001), and up to 6/10 days with enriched microbial communities in soil microcosms (Jacquiod *et al.*, 2013). This underlines the fact that degradation of recalcitrant molecules is globally not immediate, as time is needed to initiate the metabolic switch to produce required enzymes and start to tackle these complex structures.

**Figure 5:**
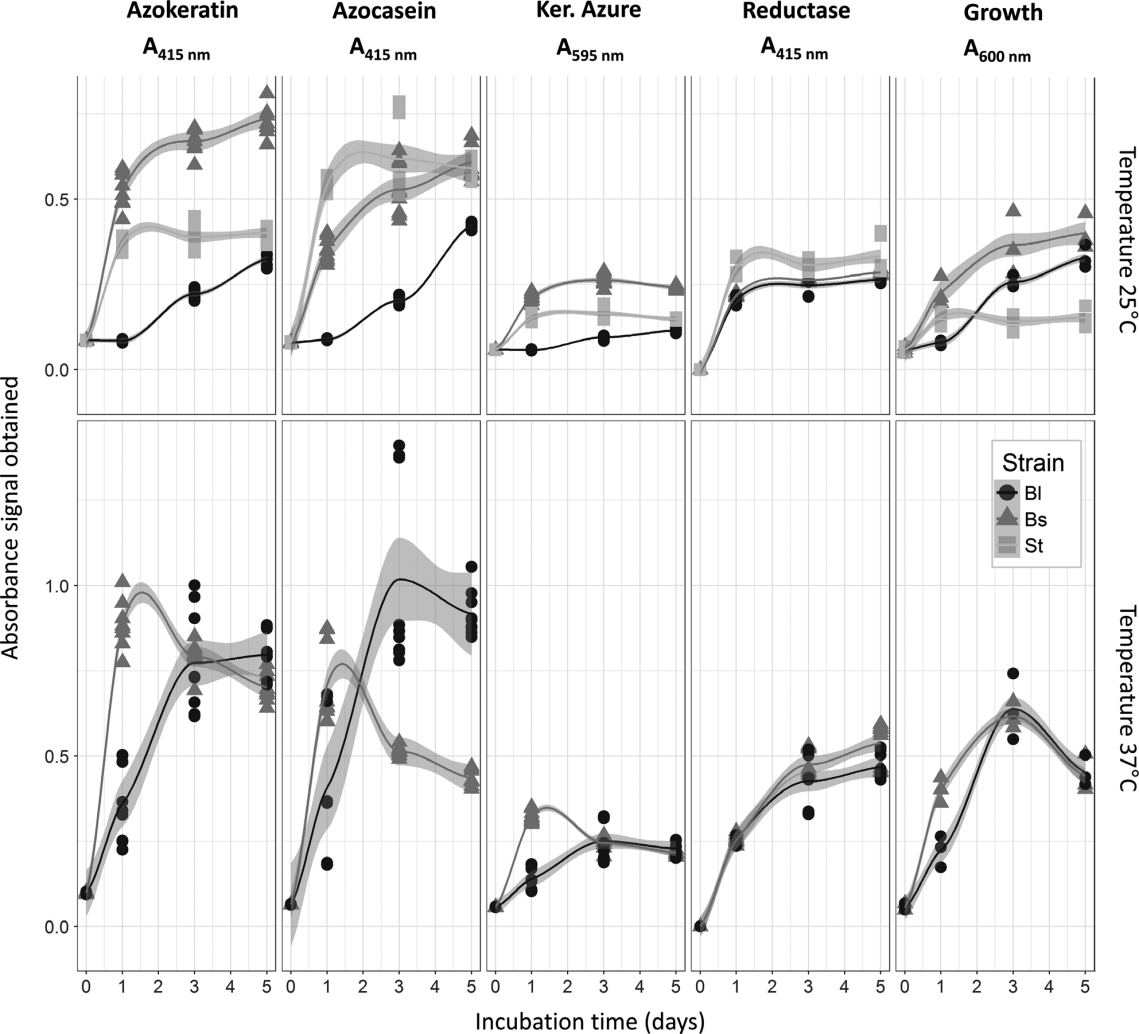
Absorbance recordings of all assays (AK: Azokeratin, 415 nm; AC: Azocasein, 415 nm; KA: Keratin Azure, 595 nm; DR: Disulphide reductase, 415 nm; Microbial growth at 600 nm) for 25°C and 37°C using *Bacillus licheniformis* (Bl), *Bacillus subtilis* (Bs) and *Stenotrophomonas* spp. (St). Grey area represents the 95% confidence intervals.

Unlike *Bacillus* strains, *Stenotrophomonas* sp. (*St*) showed a very stable and constant profile in terms of growth and enzymatic activities over time at 25°C, performing better than *Bl* at day five but being outperformed by *Bs* already at day one. The fact that *St* failed to grow at 37°C confirms its mesophilic range preference (Saba *et al.*, 2012), while its performance at 25°C suggests that its activity is stable at mesophilic temperature range. Nevertheless, this strain growth was still limited despite evidence of keratin usage from enzymatic data (Fig. 5). This strain was originally the dominant isolate retrieved from successive liquid culture enrichments inoculated with the microbial community of a moth’s gut, and using pig keratin as the sole source of nutrient (Fig. S1). The final culture batch where *St* was isolated was an enriched microbial consortium that displayed higher growth levels (Fig. S1, g6) than achieved by the *St* strain alone (Fig. 5). Thus, these observations reinforce the idea that *St* is an efficient producer of enzymes involved in keratin degradation while having difficulties to achieve growth, thus indicating that the original properties observed in the enriched consortium were likely the deeds of several cooperating species interacting together (Kang *et al.*, 2018). These observations also make sense considering the presence of different protease‐encoding genes in our three tested strains (Fig. S7; Table S1). Indeed, it has been previously shown that *Stenotrophomonas* and *Bacillus* strains proteases belong to different families with specific catalytic mechanisms (Yamamura *et al.*, 2002; Ferrareze *et al.*, 2016), thus making them potentially complementary if applied together. Therefore, if grown successfully with the right condition and partners, the remarkable mesophilic keratinase activity of *St* may be potentially interesting for saving energy purposes (Ferrareze, Correa and Brandelli, 2016). For instance, it has been suggested to include glucose during the incubation period to help *Stenotrophomonas* species growth and optimize keratinase production (Fang *et al.*, 2013).

**Figure 6:**
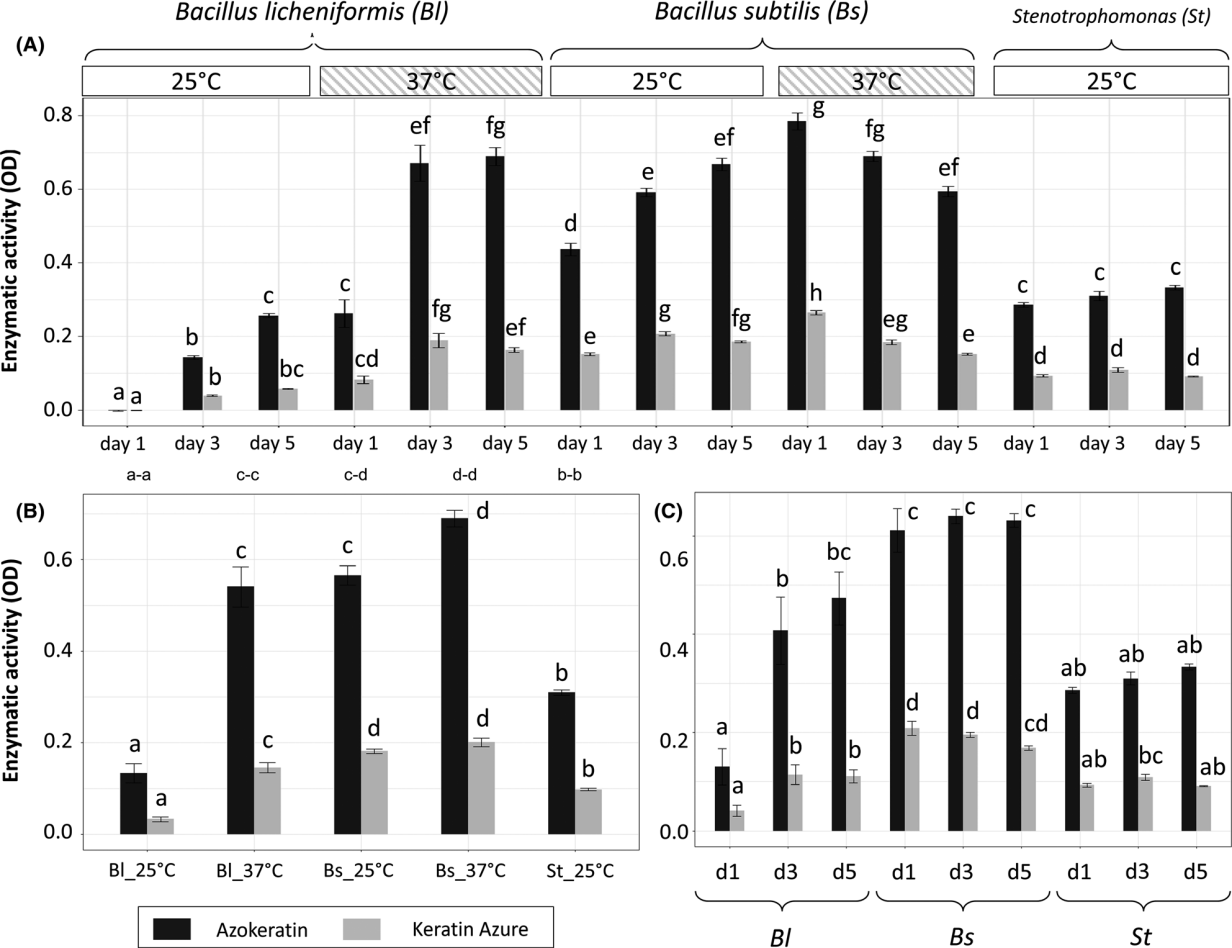
Multiple comparison test of absorbance obtained with Azokeratin (AK) and Keratin Azure (KA). Panel A shows the comparison for all tested conditions separately. Other panels, respectively, display averaging of the data according to strains/temperature combinations (B) and strains/time combinations (C). The names of each condition include, respectively, the strain identifier (*Bs*: *Bacillus subtilis*, *Bl*: *Bacillus licheniformis*, *St*: *Stenotrophomonas* spp.), the day number (1: day one, 3: day three, 5: day five) and the temperatures (25°C and 37°C). Error bars represent standard error of the mean, and letters correspond to ANOVA Tukey multiple comparison test nested within each activity assay independently (*P* < 0.05).

### Comparison between Azokeratin and Keratin Azure assays

A multiple comparison test was used to analyse differences between treatments (Fig. 6A, ANOVA, *P* < 0.05). Variance partition presented contrasting patterns within the factors (Tables S2 and S3); therefore, keratinolytic assays were compared by averaging data according to temperature and days of growth respectively (Fig. 6B and C). Regardless of time and temperatures applied, a significant difference was found between both assays when averaging all data, as Azokeratin signal was 2.7‐fold higher than Keratin Azure (Azokeratin average = 0.43 ± 0.02; Keratin Azure average = 0.16 ± 6.3E−3, *t*‐test *P* < 1.0E−4). This underlines the higher capability for assaying and resolving statistically significant differences in keratinolytic activities using Azokeratin compared with Keratin Azure due to the wider signal amplitude. After averaging according to temperature for each strain (Fig. 6B), trends between assays remained similar, indicating that both methods are reliably providing the same global information. This is reinforced by the significant positive correlations with temperature achieved by both assays, although Azokeratin had a better linear fit (Pearson'*r* = 0.4, *P* < 1.0E−4) than Keratin Azure (Pearson'*r* = 0.3, *P* < 1.0E−4). Nevertheless, in the case of *Bs*, Keratin Azure lacked the detection level required to resolve significant differences between temperatures unlike Azokeratin, highlighting again its higher sensitivity. When averaging by time for each strain (Fig. 6C), activity levels were declining with Keratin Azure during the experiment while they maintained or even slightly increased with Azokeratin yet revealing again the benefit of having higher signal amplitude and sensitivity to detect activity trends that could be otherwise missed by conventional means.

Results are coherent considering what is known about the strains used in this study. *Bl* has significantly enhanced activity at higher temperature over time, confirming its optimal growth and enzyme production at thermophilic range around 50°C (Wang and Shih, 1999). On the other hand, *St* displayed a stable activity over time at a strict mesophilic range, being in accordance with reported results on representatives from the *Stenotrophomonas* genus (Fang *et al.*, 2013). In the case of *Bs*, divergent behaviours where obtained depending on temperature. Indeed, at 25°C, Azokeratin activity increased and Keratin Azure varied slightly while on the other hand, at 37°C, both activities clearly declined despite being amongst the highest values recorded. These differences could be due to the very strong activity levels recorded after the first day, leading to a lack of available substrate over time. These different patterns could also indicate that conditions were suboptimal for *Bs* during the first incubation day (Cai and Zheng, 2009). Another explanation could be that *Bs* may have several degradation behaviours associated to growth conditions, with different enzymes being induced depending on time and temperatures (Wang and Shih, 1999). Overall, to evaluate the capacity of an assay using biological samples, it is important to consider the ability of the bacteria to use the protein substrate, the growth conditions such as time and temperature used and the different enzymes produced over the experiment.

## Conclusion

The Azokeratin procedure implemented to measure microbial keratinolytic activity gave robust and coherent results compared with the popular Keratin Azure method, being more sensitive (3‐fold increase). For both Azokeratin and Keratin Azure assays, the filtration method was more suitable for stopping reactions, circumventing several practical and scientific adverse consequences noticed upon TCA usage. Here, it was demonstrated that proteinase K can be used to efficiently standardize the signal gained from a given batch of Azokeratin to avoid unwanted variations, thus enabling improved reproducibility and comparison between studies. Practically, we verified that higher signal amplitude of Azokeratin allowed more sensitive detection of differential microbial keratinolytic behaviours linked to important growth parameters. It was also shown that using complementing assays such as disulphide reductase together with an appropriate keratinase assay may turn useful when assessing microbial keratinolytic capacities. We encourage implementation of this Azokeratin assay procedure to enable more accurate and sensitive estimation of microbial keratinolytic activities, in particular for bioprospecting purposes aiming at finding new and efficient biologically driven ways to convert recalcitrant keratin wastes *via* novel strains and/or synergistic microbial consortia.

## Experimental procedure

### Azokeratin preparation from keratin by‐products

Raw bristles and hooves from Danish Crown pig slaughterhouses were milled into a fine powder (Daka Sarval, Løsning, Denmark) used as source of carbon and nitrogen for cultures and enzyme production, and for preparation of the Azokeratin substrate by an adapted version of an azoalbumin labelling protocol (Tomarelli *et al.*, 1949). Briefly, 15 g keratin fine powder was suspended in 1 L distilled water and mixed with 100 ml 1.19 M NaHCO3 with constant stirring. At the same time, 8.65 g sulfanilic acid (Sigma‐Aldrich) was dissolved in 200 ml 0.12 M NaOH, then added 1.7 g NaNO2, 10 ml 5.0 M HCl and 10 ml 5.0 M NaOH during stirring. This solution was mixed with the milled by‐product suspension, stirred 10 min and vacuum filtered using filter paper. Azokeratin was washed with distilled water overnight using magnetic stirring and freeze‐dried.

### Keratin Azure preparation

Keratin Azure (azure dye modified sheep wool; Sigma) was ball‐milled into a fine powder (mini ball Mill Pulverisette 23, FRITSCH), at 20 oscillations per second to reach the same particle size as for the Azokeratin. Particle size was evaluated to be ~ 100 µm long through microscopic observation (Fig. S6).

### Preparation and titration of products released from Azokeratin and Keratin Azure

Supernatants containing azo‐dyed products (peptides/polypeptides) were obtained by incubating 1 ml of 10 µg ml^−1^ proteinase K and 10 mg ml^−1^ Azokeratin in 50 mM Tris, pH 8, 18 h at 37°C with shaking (1000 rpm). Azokeratin was removed by centrifugation (10 000 *g* for 10 min at 4°C) and absorbance spectra of the Azokeratin products in the supernatant were recorded (350‒500 nm; 1 cm quartz cuvette; Agilent Cary 8453 UV‐Visible Spectrometer System) after mixing 1:1 (vol/vol) with either 250 mM citrate (pH 4.0, 5.0 and 6.0), Tris (pH 7.0, 8.0 and 8.5), glycine (pH 9.0, 9.5, 10.0 and 10.5) or phosphate (pH 11.0, 11.5 and 12.0) buffer of varying pH. Absorbance of Keratin Azure products was measured at 595 nm. Samples were centrifuged (10 000 *g* for 10 min at 4°C) to remove precipitate before absorbance analysis. The titration model is derived from the Hill equation (Webb *et al.*, 2011).

### Azokeratin and Keratin Azure treatment with TCEP, proteinase K and proteases from *A. keratinophila*

Supernatants containing Azokeratin and Keratin Azure degradation products were obtained by incubating 10 mg ml^−1^ of the substrates with either (i) 1 mM TCEP or (ii) 1 µg ml^−1^ proteinase K. The proteinase K and TCEP concentrations were chosen as they enabled to optimize signal output within a reasonable timeframe. TCEP was chosen for its strong reducing agent properties, and as it does not react to atmospheric oxygen as opposed to others. In addition, two proteases purified from a known keratinolytic soil actinomycete *Amycolatopsis keratinophila* subsp. *keratinophila* D2^T^ (Al‐Mussallam *et al.*, 2003) grown on the same keratin culture medium as used in this manuscript (Espersen *et al.*, 2020). The two proteases were used at concentrations resulting in a signal in the assay comparable to proteinase K. The two proteases used to have the following accession numbers, C‐like protease: gi|1020304291 and T‐like protease: gi|1020292854 and both are predicted to be serine proteases belonging to the S1 family (Espersen *et al.*, 2020). *Amycolatopsis keratinophila* subsp. *keratinophila* D2^T^ (strain number: DSM 44409) was obtained as freeze‐dried pellet (German Collection of Microorganisms and Cell Cultures, Braunschweig, Germany). Samples were incubated for 18 h at 37°C with shaking (1000 rpm). Supernatants from Azokeratin digests were harvested by filtration (0.22 µM MultiScreen Filter plates with a Durapore PVDF membrane, Merck Millipore). Blank samples were incubated with buffer instead of active agent and used for subtraction for background. To investigate TCA induced signal loss, a 100% w/v TCA solution was added to constitute 2% or 10% of the final sample volume, vortex‐mixed, incubated 10 min on ice, followed by centrifugation (15 000 g, 10 min). Absorbance of supernatants was measured at 415 nm in 96‐well plate (plate reader Powerwave XS; BioTek).

### Proteinase K activity on SPAN

Proteinase K (lyophilized proteinase K, Sigma‐Aldrich) stock (40 µM in 50 mM Tris, 1 mM CaCl_2_, pH 8) was prepared and the concentration assessed spectrophotometrically at 280 nm using a molar extension coefficient of 36 580 M^−1^ cm^−1^ (calculated using the ProtParam tool on the ExPASy server). The proteinase K stock was kept on ice. SPAN (*N*‐succinyl‐l‐phenylalanine *p*‐nitroanilide; Sigma‐Aldrich) was dissolved in 100% DMSO to a concentration of 400 mM. A solution containing 50 mM Tris, 1 mM CaCl_2,_ 4.4 mM SPAN at pH 8 (180 µl), was placed in a 96‐well plate, heated to 37°C using the microtiter plate reader followed by the addition of proteinase K stock (20 µl) and incubated 60 min at 37°C with absorbance measurement at 415 nm every 30 s and shaking between measurements. Samples were done in triplicates. The data were fitted to a linear regression to get the activity of the proteinase K stock solution. One unit (U) was defined as the amount of proteinase K yielding 0.001 absorbance units/h. The proteinase K stock contained 972 U/ml.

### Initial reaction velocity of proteinase K with Azokeratin

Progress of 5 mg ml^−1^ Azokeratin degradation by 0.972 U proteinase K was monitored to verify that measured product release represents initial velocity. Azokeratin (5 mg and 50 mM Tris, 1 mM CaCl_2_, pH 8 (450 µl) in 2 ml round‐bottom Eppendorf tubes was pre‐heated to 37°C, followed by addition of proteinase K stock (50 µl, 0.972 U). The reaction continued at 37°C under shaking (850 rpm), to keep substrate suspended in sample, for 60 min with sampling every 10 min by rapid transfer to a 96‐well filter plate (0.22 µm, MultiScreen Filter plates with a Durapore PVDF membrane; Merck Millipore) equipped with a vacuum‐manifold filtering off residual insoluble substrate. Filtrates (200 µl) were collected in a 96‐well plate and transferred to a new 96‐well plate for absorbance measurement at 415 nm (plate reader). Time points were measured in duplicates. Data were fitted to a linear curve regression to make sure that degradation velocity was linear within the relevant time range.

### Azokeratin and proteinase K concentration dependent degradation

To decide on appropriate Azokeratin and Proteinase K concentrations, first, the effect of 0.972 U proteinase K on varying Azokeratin concentrations (2–60 mg ml^−1^) was followed. Values reported are from single sample measurements. Second, Proteinase K (0.0972–0.972 U) was used with 25 mg ml^−1^ Azokeratin in 50 mM Tris, 1 mM CaCl_2_, pH 8. Samples in triplicate were prepared as above and incubated (60 min, 37°C) on a thermoshaker and vortex‐mixed (850 rpm). Data were fitted to a linear regression model.

### Strains used for keratinase activity testing

Three strains displaying protease/keratinase activity were used for assay benchmarking: (i) *Bacillus licheniformis* PWD‐1 (ATCC^®^ 53757) (*Bl*) as an excellent feather degrading microorganism; (ii) unknown Gammaproteobacteria isolate, *Stenotrophomonas* spp. (*St*) from a private collection showing strong keratinolytic activity (this study); and (iii) *Bacillus subtilis 216* (*Bs*) environmental strain isolated from sandy soil samples from the River Sava, Slovenia (kindly provided by Ines Mandic‐Mulec, University of Ljubljana, Biotechnical Faculty, Ljubljana, Slovenia).

### Growth conditions and enzyme induction

A single colony of each strain (*Bs*, *Bl* and *St*) was transferred individually from LB agar to 5 ml Luria–Bertani medium (glass tube) and incubated overnight at 25°C shaking (200 rpm) until OD_600nm_ 0.6–0.8. Then, cultures were centrifuged (8000 *g*, 10 min) and cell pellets were washed using NaCl solution (0.9 g l^−1^). Subsequently, to induce keratinolytic activity, 1 ml of standardized culture volumes at OD_600nm_ 0.7 were mixed with 10 ml Keratin Liquid Medium (KLM) of (per litre): 0.5 g NH_4_Cl, 0.5 g NaCl, 0.3 g K_2_HPO_4_, 0.4 g KH_2_PO_4_, 0.1 g MgCl_2_.6H_2_O and 10 g milled bristles and hooves and adjusted to pH 7.5 (Lin *et al.*, 1992). Then, three technical replicates were prepared for each time point on a thermoshaker (200 rpm) for 1, 3 and 5 days at 25 °C and 37°C, as well for the negative controls which consisted of KLM alone. After each time point, KLM cultures were incubated for 5 min at room temperature to let big particles sink to the bottom. Then, 5 ml of culture was removed to measure OD_600nm._ Since incubation of KLM might release small particles, negative controls were subtracted for all samples at each time point. The supernatant containing crude enzyme was isolated by centrifugation (15 000 *g*, 10 min) and stored at 4°C. In parallel, pure cultures were checked for potential contaminations *via* colony growth selection on Keratin Agar plates as previously described (Gonzalo *et al.*, 2016).

### In situ assays: Azocasein, Azokeratin, Keratin Azure and disulphide reductase

Azocasein was hydrolysed in 24 multi‐well plates as described (Jahan *et al.*, 2010). Briefly, 1% Azocasein (Sigma) in 50 mM Tris‐HCl, pH 8.0 (800 μl) was incubated with culture supernatant (500 μl) for 1 h at 25°C with shaking (200 rpm). The reaction was stopped by 1.0 ml 10% TCA, kept 15 min at 4°C and centrifuged (4.000 *g*, 10 min) to pellet larger particles. Then, 1 ml supernatant was mixed with 1 ml 0.5 N NaOH and the absorbance measured at 415 nm (ELISA reader; Powerwave XS, BioTek). Keratinase activity was assayed using a modified protocol (Riffel *et al.*, 2003) in 24 multi‐well plates. Briefly, KLM culture supernatant (500 μl) was incubated with 10 g l^−1^ Azokeratin or 10 g l^−1^ Keratin Azure in 50 mM Tris‐HCl pH 8.0 (800 µl) for 1 h at 25°C or 37°C with shaking (200 rpm). The reaction mixture was stopped by filtration (0.22 µm, MultiScreen Filter plates; Merck Millipore). The absorbance for Azokeratin was measured at 415 nm, and at 595 nm for Keratin Azure. Disulphide reductase was assayed using Ellman's reagent, with 10 mg of 5,5′‐dithiobis(2‐nitrobenzoic acid) dissolved in 250 μl of dimethyl sulfoxide (Ellman, 1959). The buffer used consisted of 40 mM sodium phosphate, 2 mM EDTA, pH 7.6. In a 96‐well plate, 72 μl supernatant were mixed with 126 μl buffer and 2 μl Ellman's reagent. The solution was incubated for 5 min at room temperature and absorbance was measured at 415 nm as above. In all *in situ* assays, negative controls were treated with Tris‐HCl buffer instead. The final measurements were calculated as average (±SEM) of values obtained with three biological replicates from the starter culture, each done with three technical replicates (*n* = 9).

### Statistical analysis

Statistical analyses were performed on R software (R Core Team, 2017). A multiple comparison test was used to analyse differences between Azokeratin and Keratin azure efficiencies (ANOVA, Tukey's HSD Multiple Comparison post hoc test). Different letters indicate significant differences (*P* < 0.05) while similar letters indicate no differences. Correlation tests were used to infer links between tested variable using Pearson’s correlation coefficient (*P* < 0.05).

## Conflict of interests

The authors declare no conflict of interest regarding the results of this research.

## Supporting information


**Table S1.** Variance partition with an additive four‐way ANOVA
**Table S2.** Variance partition with an additive and time‐nested four‐way ANOVA.
**Table S3.** Full listing of known keratinases and proteases in tested genomes.
**Fig. S1.** Origin of the *Stenotrophomonas spp.* strain.
**Fig. S2.** Absorbance of Keratin Azure degradation products at pH 3‐8.
**Fig. S3.** Signal losses observed under different treatments and conditions.
**Fig. S4.** Signal losses depending on TCA concentrations in final samples.
**Fig. S5.** Standardization of keratinolytic activity using proteinase K.
**Fig. S6.** Microscopic observation of Keratin Azure (A) and Azokeratin (B) labelled particles.
**Fig. S7.** Repartition of keratinases and proteases between tested genomes.
