# Supplementary material for: Azo dying of α‐keratin material improves microbial keratinase screening and standardization
Source: Microb Biotechnol. 2020 Feb 28;13(4):984–96. doi: 10.1111/1751-7915.13541 (PMC7264887; doi:10.1111/1751-7915.13541)
Supplement: Supplementary file 1 — Table S1. Variance partition with an additive four‐way ANOVA Table S2. Variance partition with an additive and time‐nested four‐way ANOVA. Table S3. Full listing of known keratinases and proteases in tested genomes. Fig. S1. Origin of the Stenotrophomonas spp. strain. Fig. S2. Absorbance of Keratin Azure degradation products at pH 3‐8. Fig. S3. Signal losses observed under different treatments and conditions. Fig. S4. Signal losses depending on TCA concentrations in final samples. Fig. S5. Standardization of keratinolytic activity using proteinase K. Fig. S6. Microscopic observation of Keratin Azure (A) and Azokeratin (B) labelled particles. Fig. S7. Repartition of keratinases and proteases between tested genomes. [file MBT2-13-984-s001.docx]

**Supporting Information**

**Azo-dying of α-keratin material improves microbial keratinase screening and standardization**

Milena Gonzalo^1,#a,¶^, Roall Espersen^2,¶^, Waleed A. Al-Soud^1^, Francesco Cristiano Falco^3^, Per Hägglund^2,#c^, Søren J. Sørensen^1^, Birte Svensson^2^, Samuel Jacquiod^1, #b,*^

^1^Section of Microbiology, University of Copenhagen, 2100 Copenhagen, Denmark

^2^Department of Biotechnology and Biomedicine, Technical University of Denmark, 2800 Lyngby, Denmark

^3^Department of Chemical and Biochemical Engineering, Technical University of Denmark, 2800 Lyngby, Denmark

^#a^ Present address: Interactions Arbres/Micro-organismes, INRA/Univ. de Lorraine, Champenoux, France

^#b^ Present address: Agroécologie, AgroSup Dijon, INRA, Univ. Bourgogne Franche-Comté, France

^#c^ Present address: Department of Biomedical Sciences, Panum Institute 12.6, University of Copenhagen, Copenhagen, Denmark

^¶^ These authors contributed equally to this work.

* [samjqd@gmail.com](mailto:samjqd@gmail.com) Tel: +45 [5182 7021](tel:51%2082%2070%2021)

**List of supporting information**

**Table S1: Full listing of known keratinases and proteases in tested genomes.**

**Table S2: Variance partition with an additive four-way ANOVA**

**Table S3: Variance partition with an additive and time-nested four-way ANOVA.**

**Figure S1: Origin of the *Stenotrophomonas spp.* strain**

**Figure S2**: **Absorbance of Keratin Azure degradation products at pH 3-8.**

**Figure S3: Signal losses observed under different treatments and conditions.**

**Figure S4: Signal losses depending on TCA concentrations in final samples.**

**Figure S5: Standardization of keratinolytic activity using proteinase K.**

**Figure S6: Microscopic observation of Keratin Azure (A) and Azokeratin (B) labelled particles.**

**Figure S7: Repartition of keratinases and proteases between tested genomes.**

| **Functional subsystem** | **Description** | **Strain** |
| --- | --- | --- |
| Metallocarboxypeptidases (EC 3.4.17.-) | Thermostable carboxypeptidase 1 (EC 3.4.17.19) | Bl |
| Omega peptidases (EC 3.4.19.-) | Acylamino-acid-releasing enzyme (EC 3.4.19.1) | Bs/Bl |
| Aminopeptidases (EC 3.4.11.-) | Aminopeptidase S (Leu, Val, Phe, Tyr preference) (EC 3.4.11.24) | Bs/Bl |
| Aminopeptidases (EC 3.4.11.-) | Aminopeptidase Y (Arg, Lys, Leu preference) (EC 3.4.11.15) | Bs/Bl |
| Protein degradation | Aminopeptidase YpdF (MP-, MA-, MS-, AP-, NP- specific) | Bs/Bl |
| Proteolysis in bacteria, ATP-dependent | ATP-dependent Clp protease, ATP-binding subunit ClpC | Bs/Bl |
| Proteasome bacterial | ATP-dependent protease La (EC 3.4.21.53) LonB Type I | Bs/Bl |
| Protein degradation | Deblocking aminopeptidase (EC 3.4.11.-) | Bs/Bl |
| Omega peptidases (EC 3.4.19.-) | Gamma-D-glutamyl-meso-diaminopimelate peptidase (EC 3.4.19.11) | Bs/Bl |
| Serine endopeptidase (EC 3.4.21.-) | Glutamyl endopeptidase precursor (EC 3.4.21.19), blaSE | Bs/Bl |
| Proteasome bacterial | Lon-like protease with PDZ domain | Bs/Bl |
| Proteolysis in bacteria, ATP-dependent | Membrane protein with RNA-binding TRAM & ribonuclease PIN domains | Bs/Bl |
| Metallocarboxypeptidases (EC 3.4.17.-) | Muramoyltetrapeptide carboxypeptidase (EC 3.4.17.13) | Bs/Bl |
| Proteolysis in bacteria, ATP-dependent | Nucleotide excision repair protein, with UvrB/UvrC motif | Bs/Bl |
| Proteolysis in bacteria, ATP-dependent | Putative ATP:guanido phosphotransferase YacI (EC 2.7.3.-) | Bs/Bl |
| Omega peptidases (EC 3.4.19.-) | Pyrrolidone-carboxylate peptidase (EC 3.4.19.3) | Bs/Bl |
| Proteolysis in bacteria, ATP-dependent | Transcriptional regulator CtsR | Bs/Bl |
| Proteasome bacterial | ATP-dependent Clp protease ATP-binding subunit ClpX | Bs/Bl/St |
| Proteolysis in bacteria, ATP-dependent | ATP-dependent Clp protease ATP-binding subunit ClpX | Bs/Bl/St |
| Proteasome bacterial | ATP-dependent Clp protease proteolytic subunit (EC 3.4.21.92) | Bs/Bl/St |
| Proteolysis in bacteria, ATP-dependent | ATP-dependent Clp protease proteolytic subunit (EC 3.4.21.92) | Bs/Bl/St |
| Proteasome bacterial | ATP-dependent hsl protease ATP-binding subunit HslU | Bs/Bl/St |
| Proteolysis in bacteria, ATP-dependent | ATP-dependent hsl protease ATP-binding subunit HslU | Bs/Bl/St |
| Proteasome bacterial | ATP-dependent protease HslV (EC 3.4.25.-) | Bs/Bl/St |
| Proteolysis in bacteria, ATP-dependent | ATP-dependent protease HslV (EC 3.4.25.-) | Bs/Bl/St |
| Proteasome bacterial | ATP-dependent protease La (EC 3.4.21.53) Type I | Bs/Bl/St |
| Proteolysis in bacteria, ATP-dependent | ATP-dependent protease La (EC 3.4.21.53) Type I | Bs/Bl/St |
| Aminopeptidases (EC 3.4.11.-) | Cytosol aminopeptidase PepA (EC 3.4.11.1) | Bs/Bl/St |
| Metallocarboxypeptidases (EC 3.4.17.-) | D-alanyl-D-alanine carboxypeptidase (EC 3.4.16.4) | Bs/Bl/St |
| Proteolysis in bacteria, ATP-dependent | DNA repair protein RadA | Bs/Bl/St |
| Proteasome bacterial | Site-specific tyrosine recombinase | Bs/Bl/St |
| Proteolysis in bacteria, ATP-dependent | ClpB protein | Bs/St |
| Protein degradation | Arginine-tRNA-protein transferase (EC 2.3.2.8) | St |
| Protein degradation | Asp-X dipeptidase | St |
| Proteolysis in bacteria, ATP-dependent | ATPase, AFG1 family | St |
| Protein degradation | Dipeptidyl carboxypeptidase Dcp (EC 3.4.15.5) | St |
| Putative TldE-TldD proteolytic complex | FIG138315: Putative alpha helix protein | St |
| Omega peptidases (EC 3.4.19.-) | Isoaspartyl aminopeptidase (EC 3.4.19.5) | St |
| Protein degradation | Leucyl/phenylalanyl-tRNA--protein transferase (EC 2.3.2.6) | St |
| Protein degradation | Oligopeptidase A (EC 3.4.24.70) | St |
| Aminopeptidases (EC 3.4.11.-) | Peptidase B (EC 3.4.11.23) | St |
| Serine endopeptidase (EC 3.4.21.-) | Prolyl endopeptidase (EC 3.4.21.26) | St |
| Putative TldE-TldD proteolytic complex | TldE protein, part of TldE/TldD proteolytic complex | St |
| Proteasome bacterial | Uncharacterized protein, similar to Lon protease N-terminal domain | St |
| Aminopeptidases (EC 3.4.11.-) | Xaa-Pro aminopeptidase (EC 3.4.11.9) | St |

**Table S1: Full listing of known keratinases and proteases in tested genomes**. The list contains all identified coding DNA sequences from genomes of the three strains matching keratinases and proteases. Functions are predicted based on the Rapid Annotation System Technology (RAST) using the SEED hierarchical classification.

| **Factors tested** | **AK** | **AC** | **KA** | **MG** | **DR** |
| --- | --- | --- | --- | --- | --- |
| Temperature | 27.59 | 16.09 | 22.40 | 32.77 | 12.53 |
| Time (days) | 60.92 | 77.67 | 56.68 | 61.66 | 85.62 |
| Strain | 10.31 | 4.28 | 18.89 | 4.46 | 1.06 |
| Biological Replication | 1.18 | 1.95 | 2.02 | 1.10 | 0.79 |
| Residuals | < 0.01 | < 0.01 | 0.02 | < 0.01 | < 0.01 |

**Table S2: Variance partition with an additive four-way ANOVA**. The table shows the variance partition between all the tested parameters in percentage. All tested factors had very high significance (n = 9, p < 2.0E-16, ***). AK: Azokeratin, AC: Azocasein, KA: Keratin Azure, MG: microbial growth measured at 600 nm, DR: Disulphide reductase.

| **Time** | **Factors tested** | **AK** | **AC** | **KA** | **MG** | **DR** |
| --- | --- | --- | --- | --- | --- | --- |
| Day 1 | Temperature | 46.90 | 44.47 | 40.67 | 68.99 | 9.22 |
|  | Strain | 52.36 | 52.06 | 58.75 | 30.14 | 87.72 |
|  | Biological Replication | 0.71 | 3.46 | 0.56 | 0.87 | 2.92 |
|  | Residuals | 0.03 | 0.01 | 0.02 | < 0.01 | 0.14 |
| Day 3 | Temperature | 81.54 | 71.12 | 59.97 | 93.16 | 94.69 |
|  | Strain | 15.16 | 14.95 | 30.95 | 5.93 | 3.54 |
|  | Biological Replication | 3.26 | 13.92 | 8.94 | 0.91 | 1.77 |
|  | Residuals | 0.04 | 0.01 | 0.13 | < 0.01 | < 0.01 |
| Day 5 | Temperature | 77.07 | 47.95 | 58.72 | 63.78 | 94.90 |
|  | Strain | 16.83 | 28.46 | 33.42 | 34.50 | 3.88 |
|  | Biological Replication | 6.06 | 23.51 | 7.79 | 1.72 | 1.23 |
|  | Residuals | 0.04 | 0.08 | 0.08 | < 0.01 | < 0.01 |

**Table S3: Variance partition with an additive and time-nested four-way ANOVA.** Three independent ANOVA were done for each time point. The table shows the variance partition between all the tested parameters in percentage. All tested factors had very high significance (n = 9, p < 2.0E-16, ***). AK: Azokeratin, AC: Azocasein, KA: Keratin Azure, MG: Microbial Growth measured at 600nm, DR: Disulphide reductase.


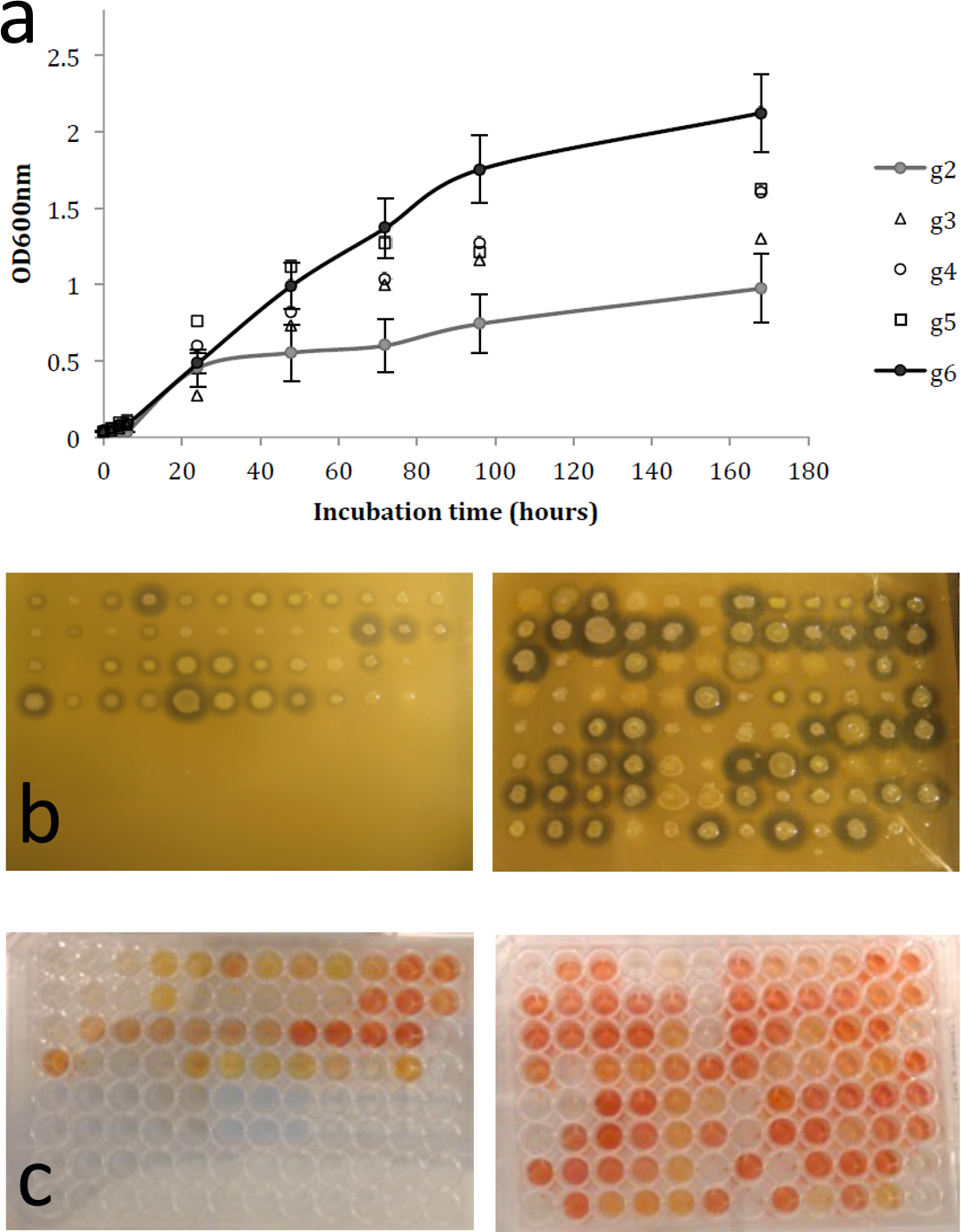


**Figure S1: Origin of the *Stenotrophomonas spp.* strain**. Panel a shows the serial enrichment procedure done at 25°C with six consecutive batches of pig alpha keratin medium initially inoculated with several complex microbial starters (e.g. soil, moth guts, wastewater…). In panel a, only batches from generation g2 to g6 are shown, with standard error of the mean error bars for g2 and g6. Panel b and c are respectively showing the proteolytic isolates recovered from g2 (left) and g6 (right), respectively on milk agar (b) and azocasein (c). *Stenotrophomonas spp*. isolates, which can be identified with the clearing halos on agar( panel b) and deep orange color (panel c), were dominating in the last generation (g6, right) which showed the most efficient consortium growth on keratin as the sole source of nutrient (a).


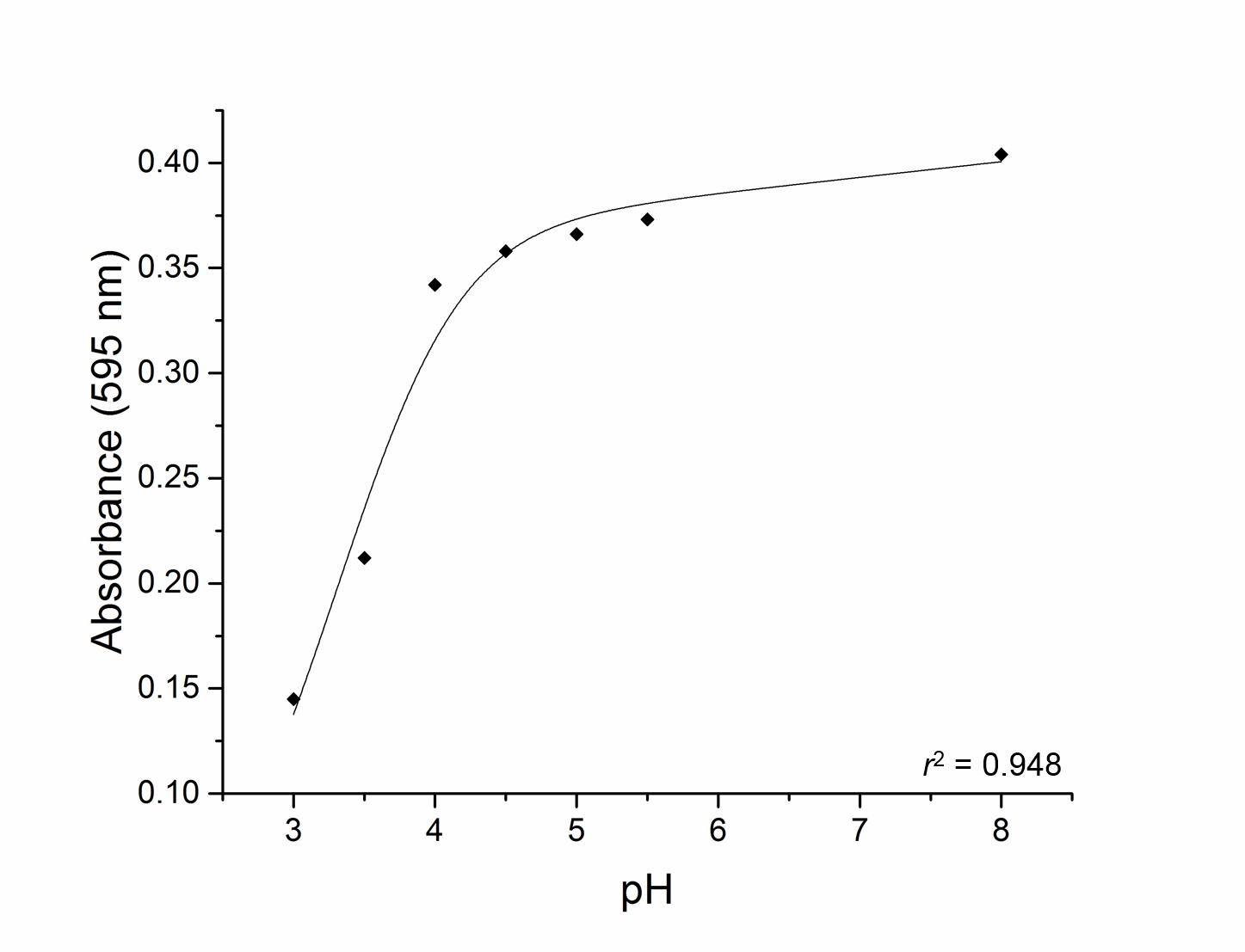
**Figure S2: Absorbance of Keratin Azure degradation products at pH 3−8.** The signal from Keratin Azure degradation product depends highly on pH. At pH < 4 the absorbance of the supernatant after centrifugation is drastically lowered. Measurements were made at 595 nm.

**
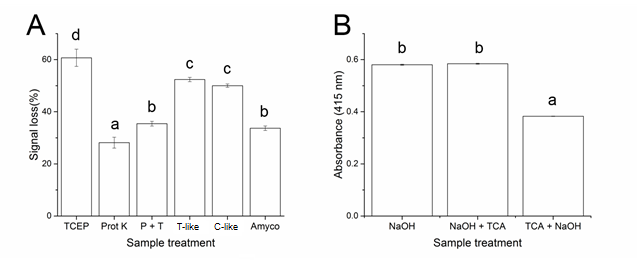
**

**Figure S3: Signal losses observed under different treatments and conditions.** Panel A shows the relative signal loss from precipitated degradation products for each treatment method. The relative signal loss for the different treatments is significantly different, with the TCEP-treated samples (TCEP), as well as the two purified proteases from *Amycolatopsis keratiniphila*, named T- and C-like protease (T-like and C-like), having high signal losses.  Moreover, the signal loss seen with the two purified proteases (T- and C-like protease), is significantly higher compared to proteinase K (Prot K). While the signal loss is lower for the sample treated with culture supernatant from *Amycolatopsis keratiniphila* grown on KLM media (Amyco), it is still higher than the proteinase K and proteinase K + TCEP (P + T). These results show the variability observed when assessing degradation with different proteases and using TCA-stopped reactions. Panel B displays the signal loss difference in samples with NaOH added either before or after TCA treatment. The figure shows no absorbance loss if NaOH is added before TCA (NaOH + TCA), when compared to a sample where only NaOH and buffer is added (NaOH). Any loss detected from samples where TCA is added and the sample is centrifuged prior to NaOH addition will thus likely be due to precipitated Azokeratin degradation products. Error bars show standard error of mean. Measurements were made in triplicates. Statistical analysis was done using ANOVA implemented with post-hoc Tukey test (*p* < 0.05).


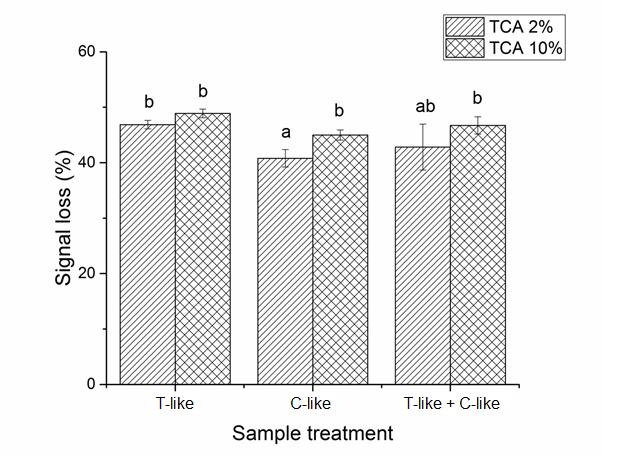


**Figure S4: Signal losses depending on TCA concentrations in final samples.** Two different TCA concentrations (2% and 10%) were tested. It can be noted that the difference in signal loss between 2% and 10% TCA is very small. This indicates that the main source of signal loss is indeed due to the mere presence of TCA. Error bars show standard error of mean. Statistical analysis was done using ANOVA implemented with post-hoc Tukey test (*p* < 0.05).


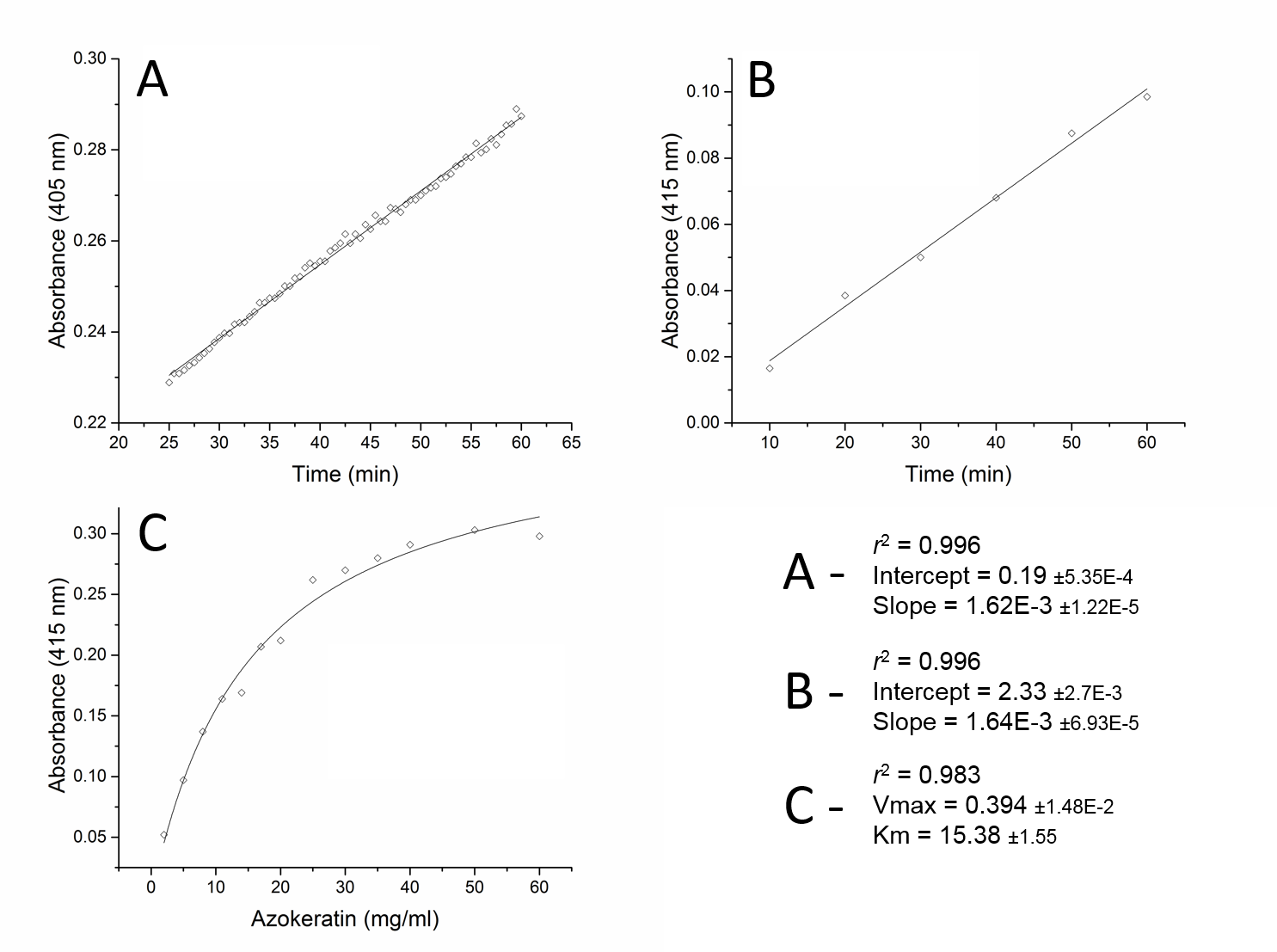


**Figure S5: Standardization of keratinolytic activity using proteinase K.** Panel A displays the monitoring of the catalytic degradation of SPAN (*N*-succinyl-L-phenylalanine *p*-nitroanilide) by proteinase K. The degradation of SPAN with proteinase K produces data which is fitted to a straight line with a slope that can be used to standardize the proteinase K activity. Panel B is showing the time dependent degradation of Azokeratin (5 mg/ml) with proteinase K (0.972 U/ml). Degradation of Azokeratin in a 5 mg/ml sample, seems to proceed at a steady rate within the measured time window. Data points are averages of two technical measurements. Panel C represents the release of Azokeratin degradation product by proteinase K at varying substrate concentration. The release of azo-product from varying concentrations of Azokeratin displays a Michaelis-Menten like shape and has thus been fitted to the Michaelis-Menten equation (*V*_max_ (Abs/h); *K*_M_ (mg/mL)).


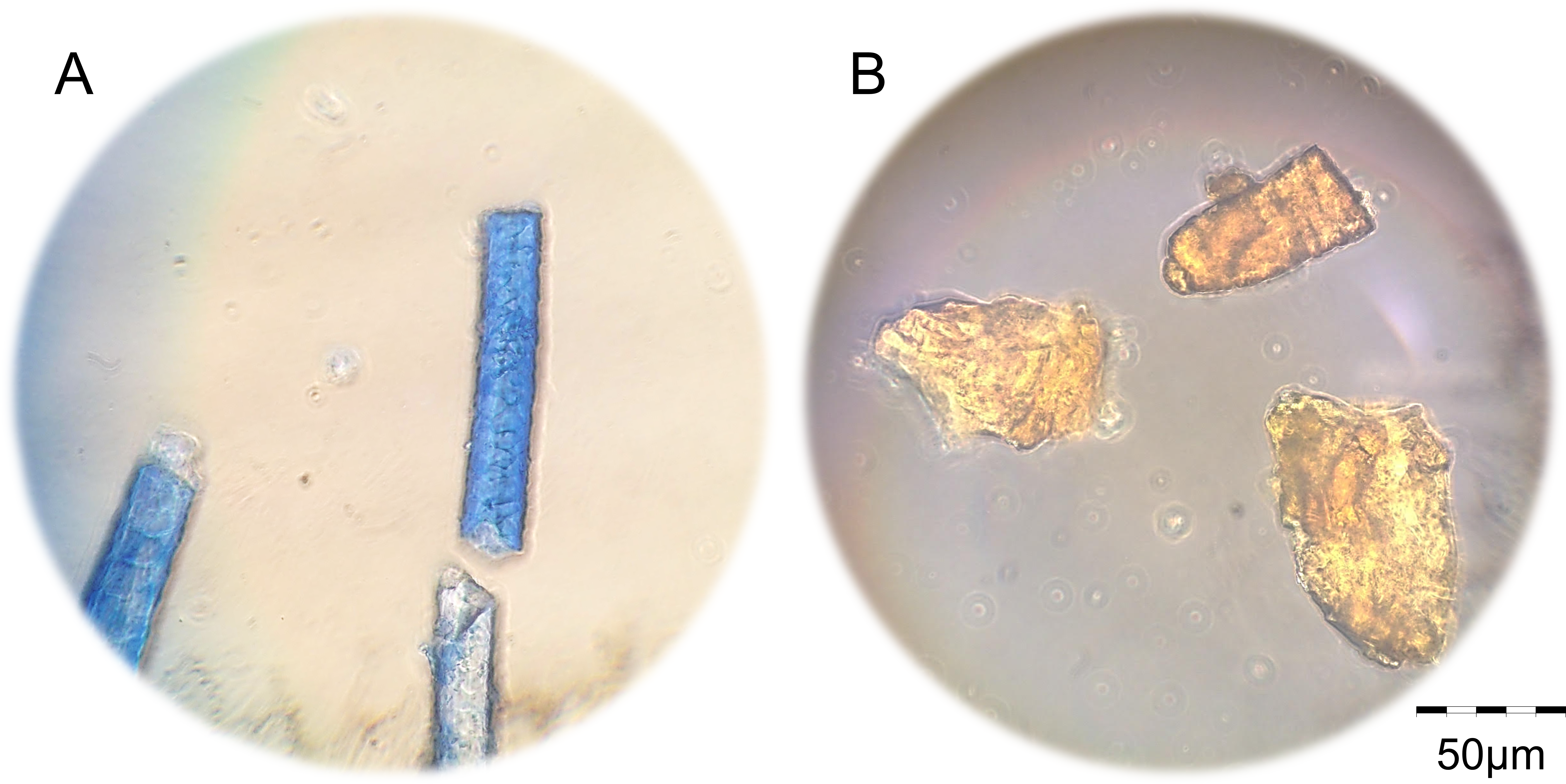


**Figure S6:** Microscopic observation of Keratin Azure (A) and Azokeratin (B) labelled particles. Pictures were taken from direct observations using and optical microscope (400x). These pictures show the shape and surface differences between the two types of substrates, with higher heterogeneity and developed surface for Azokeratin compared to Keratin Azure.

**
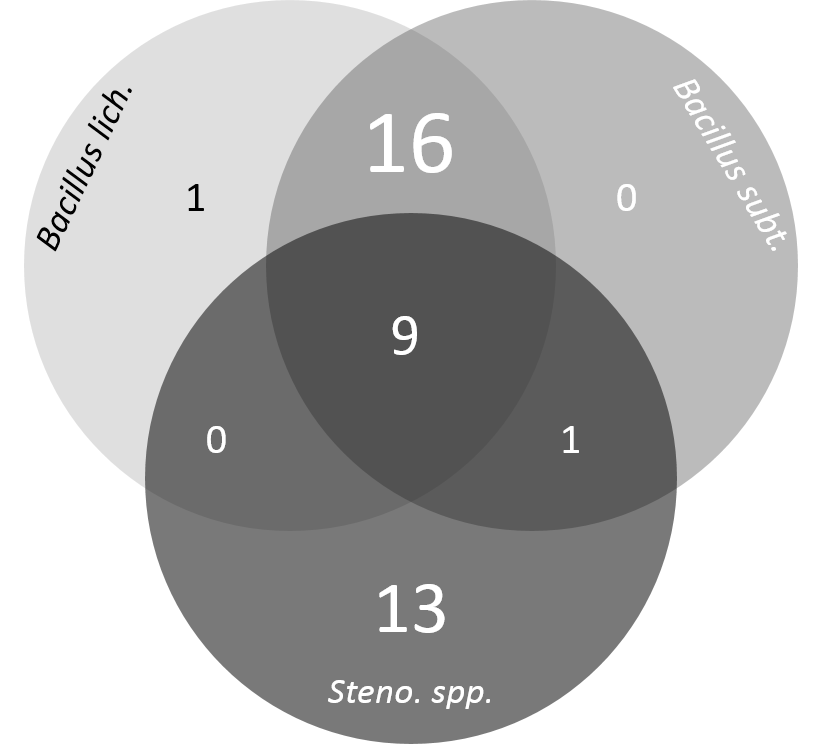
**

**Figure S7: Repartition of keratinases and proteases between tested genomes.** The figures shows a Venn diagram distribution of coding DNA sequences matching known keratinases and proteases in *Bacillus licheniformis* (*Bl*), *Bacillus subtilis* (*Bs*) and an unclassified *Stenotrophomonas spp* (*St*). The full list is provided in Table S3 with information about protein degradation function (PDF). The three strains share 31% of the PDF. *Bl* and *St* have PDF that are unique to them (2.2 and 29% respectively), in contrast with *Bs* that does not have any unique ones. *Bs* and *Bl* share 35.5% of the PDF while *Bs* and *St* only share 2%.
